# Entomopathogenic fungi and their potential for the management of *Aedes aegypti* (Diptera: Culicidae) in the Americas

**DOI:** 10.1590/0074-02760170369

**Published:** 2018-03

**Authors:** Harry C Evans, Simon L Elliot, Robert W Barreto

**Affiliations:** 1 Centre for Agriculture and Biosciences International, Egham, Surrey, UK; 2 Universidade Federal de Viçosa, Departamento de Entomologia, Viçosa, MG, Brasil; 3 Universidade Federal de Viçosa, Departamento de Fitopatologia, Viçosa, MG, Brasil

**Keywords:** *Aedes* mosquitoes, classical biological control, enemy release hypothesis, flaviviruses, infection strategies, pathogenic fungi

## Abstract

Classical biological control has been used extensively for the management of exotic weeds and agricultural pests, but never for alien insect vectors of medical importance. This simple but elegant control strategy involves the introduction of coevolved natural enemies from the centre of origin of the target alien species. *Aedes aegypti* - the primary vector of the dengue, yellow fever and Zika flaviviruses - is just such an invasive alien in the Americas where it arrived accidentally from its West African home during the slave trade. Here, we introduce the concept of exploiting entomopathogenic fungi from Africa for the classical biological control of *Ae. aegypti* in the Americas. Fungal pathogens attacking arthropods are ubiquitous in tropical forests and are important components in the natural balance of arthropod populations. They can produce a range of specialised spore forms, as well as inducing a variety of bizarre behaviours in their hosts, in order to maximise infection. The fungal groups recorded as specialised pathogens of mosquito hosts worldwide are described and discussed. We opine that similar fungal pathogens will be found attacking and manipulating *Ae. aegypti* in African forests and that these could be employed for an economic, environmentally-safe and long-term solution to the flavivirus pandemics in the Americas.

The flaviviruses have been posing threats to human health in the New World for the past four centuries, most notable has been the Yellow fever virus (YFV) (Flavivirus: Flaviviridae). It has even been conjectured that this virus influenced the balance of power and maritime geo-politics in the Americas after its arrival in the Caribbean region in the 1640s (Van der Weijden et al. 2007). Nevertheless, it could be argued that, in more recent times, Dengue virus (DENV) has far surpassed the impact of the YFV in the Americas, and is now “considered to be the most important mosquito-borne viral disease in the world” (Araújo et al. 2015a). Until recently, there were no vaccines or specific therapeutic agents to combat it (Simmons et al. 2012): a situation now changed with the report of a potential vaccine (Osorio et al. 2011), and the subsequent launch of Dengvaxia® (Sanofi-Pasteur). The global impact is immense with an estimated 50 million cases of dengue fever per year (WHO 2009); although this has been re-estimated at closer to 400 million (Bhatt et al. 2013). However, both these major human diseases have temporarily been surpassed in importance as Zika virus (ZIKV) has swept through the Americas; causing explosive pandemics (Fauci & Morens 2016), and prompting the World Health Organization to declare a Public Health Emergency of International Concern (Gulland 2016, Musso & Gubler 2016, Petersen et al. 2016, Russell 2016, Samarasekera & Triunfol 2016). World attention has focused on this latest invasive flavivirus because of its purported association with congenital neurological disorders, such as microcephaly and Guillain-Barré syndrome (Gatherer & Kohl 2016, Plourde & Bloch 2016, Wikan & Smith 2016). There is increasing and compelling evidence to confirm the supposition that the ZIKV is, indeed, the cause of these congenital defects (dos Santos & Goldenberg 2016, Mlakar et al. 2016). The principal vector of all these exotic viruses in the Americas is *Aedes aegypti* (Diptera: Culicidae): often called the yellow fever mosquito and which, together with the flaviviruses, is also an invasive alien species from Africa (Powell & Tabachnick 2013).

There are increasing calls for new strategies and products to improve the management of *Ae. aegypti* (Maciel-de-Freitas et al. 2012, Araújo et al. 2015a, Musso & Gubler 2016, Petersen et al. 2016): highlighted by the article, “Zika virus outbreak in the Americas: the need for novel mosquito control methods” (Yakob & Walker 2016). Similar calls for a broader and more integrated research approach that expands our knowledge of the complex ecosystems involved have been made (Fauci & Morens 2016), especially in the light of claims that the various control campaigns launched by the Brazilian Government appear to have had little impact and that the battle against the mosquito is being lost (Araújo et al. 2015a, Samarasekera & Triunfol 2016, Vogel 2016). Benelli et al. (2016) explored the use of biological control against mosquito vectors, including *Ae*. *aegypti*, highlighting the potential of bacteria, especially *Wolbachia*. It was concluded that: “The paucity of studies describing the effects of fungi on mosquito populations indicates further research is needed” (Benelli et al. 2016).

Here, we assess and discuss the role that entomopathogenic fungi could play in managing *Ae. aegypti* in Latin America and opine that classical biological control - and, understanding the controlling factors and the natural balance of mosquito populations in Africa that comes with this approach - is just such an innovative vector control strategy.

*The target: Ae. aegypti* - The long-held assumption that the YFV and its mosquito vector first arrived in the Americas from Africa via the slave trade - purportedly, ‘hitching a ride’ in the bilges of sailing ships - has now been confirmed using gene sequence data to calculate divergence times (Bryant et al. 2007). Based on phylogenetic analyses, the latter authors calculated that the genetic variability in the South American virus arose within the last three to four centuries, offering support that the virus, along with *Ae*. *aegypti*, was introduced during the slave-trade era, when contact between Africa and the Americas was first made. The yellow fever mosquito is also an efficient vector of Chikungunya virus (CHIKV) (Alphavirus: Togaviridae) (Diallo et al. 2012), as well as the other flaviviruses: dengue (Moore et al. 2013, Powell & Tabachnick 2013, Araújo et al. 2015a) and Zika (Diagne et al. 2015, Hennessey et al. 2016, Musso & Gubler 2016, Solomon et al. 2016). Recent studies of mitochondrial DNA from exotic populations of *Ae. aegypti* have revealed a dual African origin: a basal clade from West Africa; and, a second clade - derived from this basal clade - present in East Africa (Bracco et al. 2007, Moore et al. 2013). Two genetically-distinct subspecies have now been confirmed: *Ae*. *aegypti aegypti* and *Ae*. *aegypti formosus* (Gloria-Soria et al. 2016). These co-occur in both East Africa (Kenya) and West Africa (Senegal). In forest habitats in Kenya, where *formosus* is the dominant and ancestral form (Yalwala et al. 2015), the two subspecies remain distinct but ingress freely in urban situations.

The increase in infestations of *Ae*. *aegypti* in subtropical and tropical regions of the Americas is considered to be an important factor in the Zika pandemic, and also in the on-going dengue outbreaks (Russell 2016). Rise in mosquito populations has been linked to increased urbanisation, improved transportation and resistance to insecticides (Dusfour et al. 2011, Maciel-de-Freitas et al. 2012, Flores et al. 2013, Hotez 2016). We also consider here that the absence of coevolved natural enemies has contributed significantly to the on-going situation, which is posited to be a key factor in invasions by alien species (Keane & Crawley 2002). In fact, this is a re-invasion of South America by *Ae*. *aegypti* from foci in the Caribbean and North America, since the mosquito was mostly eradicated from the continent by the late 1960s, following almost two decades of an extensive and expensive campaign that involved both cultural (removal of breeding sites) and chemical control (Camargo 1967, Hotez 2016).

*The strategy: classical biological control* (CBC) - A major reason factored in to explain the success of invasive alien species (IAS) is the release from their natural enemies: encapsulated in the enemy release hypothesis (Keane & Crawley 2002). All species in their native ranges are host to natural enemies, either generalists or specialists: the latter have long associations with their hosts, which often generate a co-evolutionary relationship. Thus, escape from these natural enemies increases host fitness and can lead to population explosions, pest outbreaks and invasiveness. One well-established strategy for managing IAS - classical or inoculative biological control - has been to introduce host-specific or coevolved natural enemies in order to suppress the biotic invader and to restore the ecological balance that is lost when species escape their specialised natural enemies left behind in the evolutionary centre (Bellows 2001, Mitchell & Power 2003). CBC - or, “the intelligent introduction of counter-pests” (Elton 1958) - has been used extensively and, in many cases, very successfully as a management strategy for invasions by alien arthropods and plants; often, following the release of a single (‘silver bullet’) natural enemy. By far the greater majority of examples have involved insect agents: notably, parasitoids for invasive alien pests of crops (Neuenschwander 1996, Henry et al. 2010) and, insect herbivores for exotic weeds (McFadyen 1998); although, more recently, fungal plant pathogens have come to the fore (Evans 2013a). Moreover, it would not be an exaggeration to say that millions of lives and livelihoods have been saved, when subsistence and cash crops have been threatened by biotic invaders - especially in sub-Saharan Africa following the successes of the cassava mealybug, cassava green mite and the mango mealy-bug CBC programmes (Herren & Neuenschwander 1991, Bokonon-Ganta et al. 2002, Moore 2004, Yaninek 2007) - whilst natural ecosystems have been conserved or restored (Hoddle 2004, Culliney 2005).

In contrast to the use of insect biocontrol agents, the exploitation of pathogenic fungi in CBC has been restricted, mainly because the practitioners have had an overwhelmingly entomological background, but also, in part, due to regulatory issues (Maddox et al. 1992) and pathophobia (Warner 2012), especially involving fungal pathogens of plants (Evans 2013a). Nevertheless, although the use of fungal agents for the CBC of invasive alien weeds has a relatively short history - compared to insect agents - there have been some notable successes in both agricultural and natural ecosystems (Evans 2013a); whilst for invasive insect pests, there are few examples where the classical strategy involving pathogenic fungi has been employed (Evans 2003, Hajek & Delalibera 2010). Most of the history relating to the biological control of arthropod pests with fungi has involved the augmentative or inundative strategy with the development of formulated, semi-commercial or commercial products (mycoinsecticides), invariably based on generalist indigenous pathogens, most notably belonging to the genera *Beauveria* and *Metarhizium* (Jenkins et al. 1998, Inglis et al. 2001, Shah & Pell 2003, Wraight et al. 2007). And, more recently, the well-trodden inundative approach has also received a significant amount of attention for the control of mosquito pests, particularly aimed at *Anopheles* vectors of malaria (Blanford et al. 2005, Scholte et al. 2005, Hughes & Boomsma 2006, Scholte et al. 2008, Darbro et al. 2011, Blanford et al. 2012). However, the question has been posed: “Should the much cheaper option of classical biological control, so successfully exploited for weed pathogens, be more closely pursued using entomopathogens for the management of alien arthropod pests?” (Evans 2003).

*Entomopathogenic fungi as CBC agents of invasive alien arthropods* - The CBC strategy to control invasive alien arthropods with fungal pathogens - specifically, co-evolved or old-encounter associations as opposed to generalist or new-encounter associations, such as *Metarhizium anisopliae* on mosquito hosts (Butt et al. 2013) - has rarely been used (Shah & Pell 2003, Hajek 2007, Hajek et al. 2007, Hajek & Delalibera 2010); and, never against mosquitoes (Weiser 1991, Scholte et al. 2004, Rodriguéz-Pérez et al. 2014). Of the few examples, however, success has been both unexpected and sustained, particularly since the CBC agents involved were obligate fungi of the order *Entomophthorales* which are fastidious and, seemingly, difficult to exploit. The first involves: “the miraculous appearance of an entomopathogenic fungus [*Entomophaga maimaiga*], which, soon after its appearance, started to decimate gypsy moth [*Lymantria dispar*] populations. It provides a good example of the ‘enemy release hypothesis’, because high populations of this pest are not common in its areas of origin [Asia] but severe outbreaks occur in North America where gypsy moth has invaded without its natural enemies” (Hajek 2007). The convoluted history of the classical introduction, and the confusion surrounding it, has been well documented (Andreadis & Weseloh 1990, Hajek 1999, Pell et al. 2001, Hajek 2007). Suffice it to say that, with the aid of molecular tools, the virulent isolate of *E*. *maimaiga* which has kept pest populations in check originated in Japan, and far out-performed any of the other imported CBC agents: 13 insect natural enemies and one baculovirus (Hajek 2007). A similar success was achieved when an Israeli strain of *Erynia* (*Zoophthora*) *radicans* was released in Australia against the highly invasive spotted alfalfa aphid (*Therioaphis trifolii*). This aphid is no longer a major pest in Australia due, in part, to *E*. *radicans* but also aided by the introduction of a parasitic wasp (Pell et al. 2001, Shah & Pell 2003).

*Potential CBC agents of Ae. aegypti* - The weapons of choice are entomopathogenic fungi because of their reported ubiquity and apparent diversity on mosquito hosts (Roberts 1970, Couch & Bland 1985, Weiser 1991, Pell et al. 2001, Shah & Pell 2003, Scholte et al. 2004, Wraight et al. 2007); and, on arthropods in general, most notably in tropical forest ecosystems (Samson et al. 1988).

“As the use of fungi to control mosquitoes has been proposed from time to time since before the turn of the century, this approach to population control can hardly be considered a novel one today” (Roberts 1970). However, in this first review of the subject, the latter author did indicate that most of the research had been of a taxonomic, rather than a practical nature and, therefore, that this approach was still wide open for exploration. In the second review, over 30 years later, the need to continue investigating the potential of entomopathogenic fungi - which had waned, in part, due to the discovery of the mosquito-pathogenic bacterium, *Bacillus thuringiensis israelensis* - was stressed because of “continuous and increasing levels of insecticide resistance and increasing global risk of mosquito-borne diseases” (Scholte et al. 2004). Only a decade on, with the pandemic spread of the flaviviruses, this would seem to be a prescient statement which appears to have fallen by the wayside. Here, the genera of fungi that have been recorded from natural populations of mosquitoes, in general, and from the genus *Aed*es, in particular, are listed and annotated as to their CBC potential.

*Coelomomyces (Blastocladiomycota: Blastocladiales)* - These are obligatory parasitic fungi with complex alternating life cycles - involving microcrustacean heteroecious hosts - and restricted to aquatic Diptera, including genera of Culicidae (e.g. *Aedes*, *Anopheles*, *Culex*); typically, the infected ovaries of adult females are infertile (Couch & Bland 1985). Pathogenicity studies indicate that these are not host specific, although they may have restricted host ranges, and are frequently associated with epizootics. Nevertheless, as with some rust species (Evans 2013a), fungal pathogens with alternating life cycles are not considered to be suitable as CBC agents.

*Entomophthora (Entomophthoromycotina: Entomophthorales)* - A number of species are known from adult mosquitoes, including *Entomophthora culicis* and *E*. *destruens* from *Culex* species in Europe - where most insect-host records, in general, originate - and, the genus is often considered to have a predominantly Palaearctic distribution (Eilenberg 2000, Pell et al. 2001). Nevertheless, species of *Entomophthora* were also found to be common on adults of Culicidae in the tropical high forest of Ghana (Evans 1974). In total, eleven species of the genus *Entomophthora* have been linked to adult mosquitoes in the genera *Aedes*, *Anopheles* and *Culex* (Eilenberg 2000, Pell et al. 2001, Scholte et al. 2004). Thus, this genus holds some promise as a source of CBC agents of *Ae*. *aegypti*.

*Erynia* (*Entomophthoromycotina*: *Entomophthorales*) - Unusually, one species (*Erynia aquatica*) attacks both the larval and pupal stages of mosquitoes rather than just the adults, and*,* in North America it is associated with epizootics in populations of both *Aedes canadensis* and *Ae*. *cantator* (Anderson & Ringo 1969, Andreadis & Magnarelli 1983); whilst *E*. *conica* and *E*. *radicans* have been shown to infect adult *Ae*. *aegypti* in laboratory tests, with up to 100% infection (Scholte et al. 2004). Because *Erynia* has been linked directly with natural disease outbreaks on *Aedes* hosts - rather than just from lab-based pathogenicity tests - and its ability to infect all life stages, this genus has considerable potential and there is every reason to believe that host-specific *Erynia* species occur on *Ae*. *aegypti* in Africa.

*Culicinomyces* (*Ascomycota*: *Pezizales*) - *Culicinomyces clavisporus* is a facultative parasite of the larvae of a range of mosquito hosts, including *Aedes*, *Anopheles* and *Culex*, with infection of *Ae*. *aegypti* being demonstrated *in vitro* (Scholte et al. 2004). Natural infections of *Ae. kochi* by a tropical species, *C*. *bisporalis*, have been reported from rainforest in Australia, following the collection of larvae from breeding sites in the leaf axils of understorey plants (Sigler et al. 1987). In laboratory tests, larvae of *Ae*. *aegypti* proved to be highly susceptible when challenged with the fungus (Sigler et al. 1987). This genus shows exceptional potential because of its occurrence in tropical forests on *Aedes* mosquitoes, as well as its ability to grow *in vitro*, and there is a high probability that *Culicinomyces* is associated with *Ae*. *aegypti* in its natural range in Africa.

*Tolypocladium* (*Ascomycota*: *Hypocreales*) - This large genus contains several species that are facultative parasites of mosquito larvae, including *T*. *cylindrosporum* - originally isolated from *Ochlerotatus* larvae in both the USA and New Zealand - which has been shown to be highly pathogenic to larvae of *Anopheles* and *Culex* in small-scale trials (Evans 2003, Scholte et al. 2004), as well as to *Aedes triseriatus* (Nadeau & Boisvert 1994). Although this genus has not yet been recorded on *Aedes* under natural conditions, there is a possibility that it occurs on *Ae*. *aegypti* in African forest ecosystems.

It should be noted, however, that none of these genera have been isolated from *Ae*. *aegypti* in the field and, overwhelmingly, the studies to date have focused on temperate or subtropical regions in North America, Europe and Australasia - rather than from tropical Africa - and on diverse mosquito hosts. Moreover, laboratory pathogenicity tests can be misleading, in terms of host range and specificity, with frequent false positives and many records have been from experimental studies or from *ad hoc* field collections, with few, if any, field surveys targeted at the pathogenic mycobiota of mosquitoes. Also, note that the ‘favoured’ genera - traditionally employed in mycoinsecticides - have not been included here because, as observed previously (Roberts 1970): “The higher *Beauveria* and *Metarhizium* are not normally associated with mosquitoes and thus require repeated applications for use in control”. This goes against the coevolved, sustainable (self-replicating) philosophy of CBC. In fact, a recent study on the use of *Metarhizium* for biological control of mosquitoes has shown the absence of a host-pathogen response and that *Metarhizium* has not evolved a specific mechanism to interact with mosquitoes (Butt et al. 2013) - supporting the much earlier held opinion of Roberts (1970), as well as Weiser (1991) - and that mortality is stress induced. In fact, subsequent studies have shown that this applies only to the dry conidia - which, in keeping with the greater majority of entomopathogenic fungi, only infect via the cuticle - and that the slimy blastospores produced in liquid culture can infect via the cuticle and, if ingested, also via the gut; making blastospores a much more efficient inoculum form for control of mosquito larvae (Alkhaibari et al. 2016). Nevertheless, the argument still holds that although effective control may initially be achieved by inundative application of such blastospore-containing products, this would not be sustainable and, as with chemical insecticides, repeated applications would be necessary.

Recently, the use of non-specialised, exotic biocontrol agents to tackle the Zika and dengue outbreaks in Brazil has been criticised as potentially harmful to the native biodiversity (Azevedo-Santos et al. 2016). In this case, ‘mosquito fish’ - generalist feeders belonging to non-native species of the genus *Poecilia* - were being distributed within Brazil. This is the complete antithesis of the CBC strategy, which is based on the environmental safety guaranteed by using coevolved, host-specific natural enemies.

*Evidence of the potential of CBC* - “The only intervention for Zika virus is mosquito control, which, for *Aedes* mosquitoes, is notoriously difficult to sustain” (Solomon et al. 2016). Here, we put forward the case for CBC, which is founded on the premise that the coevolved natural enemy should be self-replicating once released into an exotic ecosystem; although, in urban situations where control is urgently needed or the target areas are vast -such as with the cassava mealybug programme in Africa (Herren & Neuenschwander 1991) - a highly-organised, technically-advanced, mass-production, classical-inundative strategy could also be employed. South American parasitoids of the invasive mealybug host were even encased for aerial delivery, and a much less refined mass-production, aerial application strategy was also used to control an invasive Madagascan weed with a coevolved rust fungus over vast areas of tropical Australia (Evans 2013a). This may be the case with *Aedes* in the Americas - for example, in high-risk urban systems - for which a product (biopesticide) could be developed for safe use in such priority situations, and which, potentially, could be self-sustaining once applied. The technology exists to mass-produce and formulate fungi in the *Ascomycota* (Jenkins et al. 1998, Inglis et al. 2001, Evans 2003) - such as *Culicinomyces* and *Tolypocladium* - and this approach could even be considered for certain obligate pathogens in the *Entomophthoromycotina*, since resting bodies and mycelia of some genera (e.g. *Erynia*) can be manipulated *in vitro* for field application (Shah et al. 1999).

The concept proposed here - that of using coevolved fungal pathogens - is under-pinned by the ability of these fungi to adapt to their hosts and to maximise infection by exploiting not only a range of spore types, but also the timing of spore release to coincide with host ecology: characteristics conspicuously absent in generalists, such as *Beauveria* and *Metarhizium*. For example, *Culicinomyces bisporalis* - discovered on *Aedes* larvae in a tropical rainforest (Sigler et al. 1987) - produces not only spores encased in mucilage, which may be an adaptation to an aquatic habitat, but also several other spore forms with, as yet, unknown functions in the infection cycle. Similarly, *Erynia aquatica* on larvae of *Aedes* species in floodwaters and marshes in North America exhibits different spore types (Anderson & Ringo 1969, Andreadis & Magnarelli 1983), and it is now known that the secondary spores, forcibly discharged from primary spores on larvae and pupae floating on the water, can infect newly-emerging adult mosquitoes (Samson et al. 1988, Roy et al. 2006); and, moreover, that this ‘artificial’ buoyancy is fungal-mediated. Other *Entomophthorales* - notably, aquatic *Erynia* species from temperate regions, such as *E*. *conica* on black flies (Simuliidae) - show remarkable adaptations with up to four spore types being produced by a single species to facilitate dispersal and infection, both aerially and aquatically, and revealing a great plasticity in spore morphology, as well as in mode of germination (Samson et al. 1988, Pell et al. 2001, Roy et al. 2006). Furthermore, there is evidence that entomophthoralean fungi kill their hosts at specific times in synchrony with the climatic conditions that favour development and transmission of the pathogen (Roy et al. 2006).

In a final twist, there is increasing evidence that arthropod hosts, infected and killed by species of *Entomophthorales*, exhibit increased sexual attractiveness to potential mates: in essence, necrophilia with venereal disease implications. Males of *Musca domestica*, for example, are significantly more attracted to infected females than to healthy ones, and it has been conjectured that the fungus produces semiochemicals to attract the males (Roy et al. 2006). In addition, not only do these males become infected themselves, but females after mating with such males before they die, lay fewer eggs. Recent work on spider mites attacked by the entomophthoralean fungus *Neozygites floridana* confirms that the same fatal attraction is in operation: the males preferring females killed by the pathogen; whilst also exploring cadavers surrounded by fungal spore ‘minefields’ (Trandem et al. 2015). Obviously, such bizarre behaviour promotes transmission of the fungus and maximises infection rates.

Doubtless, the species of *Entomophthorales* common on dipteran hosts and collected by us during scoping surveys in a vestige of Atlantic rainforest in Brazil - as shown in Fig. 1 - possess similar diversity in spore form and function, as well as behavioural traits, to ensure infection of their low-density hosts. Unfortunately, however, information on this fungal group is sparse for tropical ecosystems. However, it may be prescient to consider recent studies on fungi in the *Hypocreales*, parasitic on ants in Neotropical forests, which have shown not only this plasticity in spore form and function, but also a hidden diversity revealing highly specific host-pathogen interactions (Evans et al. 2011, Araújo et al. 2015b, Hughes et al. 2016). The so-called zombie-ant fungus, *Ophiocordyceps unilateralis sensu lato*, found in Atlantic rainforest (Fig. 2A-B) can have two-three asexual stages, to increase infection options, in addition to the characteristic sexual structure (ascostroma containing ascospores) emerging from the dorsal neck. The multiseptate, forcibly-released ascospores show a range of germination modes: producing secondary asexual stages (Fig. 2C); or, specialised needle-like capillisporophores with sticky spores (capillispores) at the apex (Fig. 2D-E). The capillispore stage is a remarkable example of convergent evolution, since the primary spores of some species of *Entomophthorales* also germinate to produce identical structures (Samson et al. 1988, Pell et al. 2001, Roy et al. 2006). Thus, ‘minefields’ are created on or below the vegetation on which the infected insects die, posing a threat to potential hosts randomly passing through or even specifically visiting them to oviposit or to feed (Evans et al. 2011, Araújo et al. 2015b, Hughes et al. 2016).

**Fig. 1:**
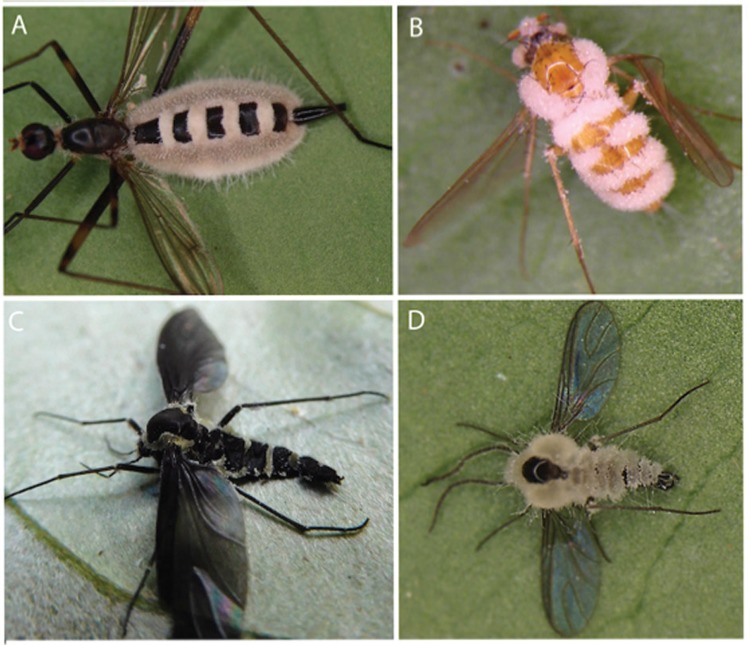
examples of *Entomophthorales* infecting Dipteran hosts; all on underside of shrub leaves resulting from several days of collecting (April 2016), in fragment of Altantic rainforest, Minas Gerais, Brazil; (A-D) all hosts show bands of fungal mycelium emerging from the body sutures, sometimes enveloping the abdomen (A) or whole body (D), producing the forcibly-released spores (ballistospores); with (A, D), or without (B, C), obvious mycelial strands (rhizoids) attaching the host to the substrate.

**Fig. 2:**
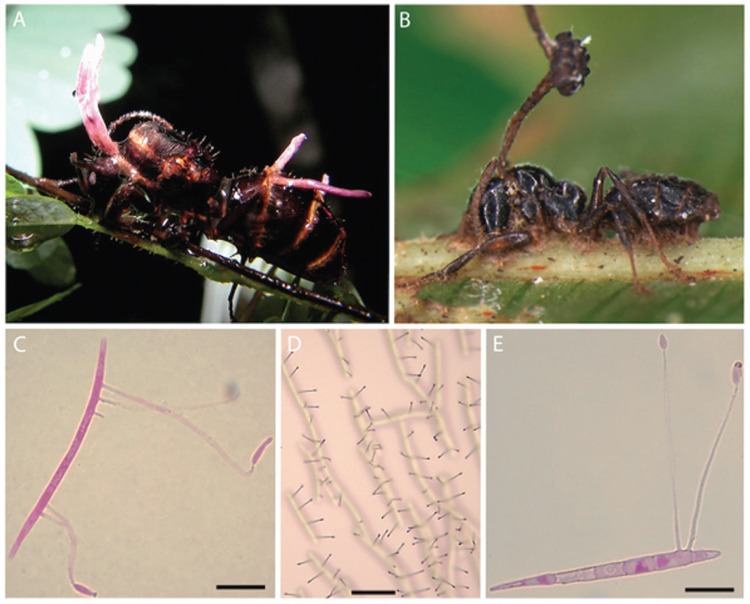
spore form and function in the zombie-ant fungus, *Ophiocordyceps unilateralis sensu lato*; all collected at the same time and from the same locality as specimens in Fig. 1; (A) zombie-ant queen of *Camponotus* species, dying with its mandibles embedded in foliage of forest shrub and immature fungal structures emerging from sutures of upper neck and abdominal regions; (B) zombie-ant worker of a different species of *Camponotus* in a similar biting position, with mature sexual stage (ascostroma) emerging; (C-E) germinating ascospores, producing specialised needle-like outgrowths (capillisporophores) with sticky capillispores, analogous to the germination process in the *Entomophthorales*; note the ascospores deposited on a glass slide, producing rows of sticky ‘traps’ to attach to passing ants (D). Bars = 10 μm, except E = 30 μm.

Finally, there is increasing evidence that arthropod hosts exhibit other behavioural changes when infected by fungal pathogens - the extended phenotype (Hughes 2013) - as, for example, in the order *Hypocreales*: the zombie ants climb shrubs and grip the foliage with mandibles and legs (Evans et al. 2011, Araújo et al. 2015b, Hughes et al. 2016) (Fig. 2A-B); ghost spiders leave their silken retreats and die on the undersides of leaves (Evans 2013b, Hughes et al. 2016). Similarly, hosts infected with *Entomophthorales* ascend vegetation (summit disease): subterranean sugar-beet aphids emerge above ground to die; carrot flies move away from the crop with the females altering their soil egg-laying habit so that they deposit their eggs on the foliage of hedgerow trees (Roy et al. 2006, Hughes et al. 2016, Gryganskyi et al. 2017). And certain genera - such as *Strongswellsea* on dipterans and *Massospora* on cicadas - even keep their infected hosts alive and the fungal spores are dispersed from body cavities during flight (Pell et al. 2001, Roy et al. 2006, Hughes et al. 2016). A recent comprehensive study of the *Entomophthorales* (COST 2007), has shown for the first time that the greater majority of species exhibit high levels of host specificity; mirroring that in the zombie-ant fungi of the *Hypocreales* (Evans et al. 2011, Araújo et al. 2015b, Hughes et al. 2016). In addition, latest research on the latter fungi has revealed that they produce a cascade of metabolites within the host - enzymes and enterotoxins - which can impact on functioning of the brain by changing the levels of neurotransmitters, such as serotonin and dopamine (de Bekker et al. 2015, Shang et al. 2015). These examples demonstrate that coevolved entomopathogenic fungi have developed a remarkable array of strategies to ensure that their spores reach the target hosts; and, furthermore, there is every reason to expect that fungi with similar traits will be found on endemic species of *Aedes*, such as *Ae*. *aegypti*, in Africa.

*Realising the potential of CBC* - The first phase would be to conduct field surveys for fungal pathogens of *Ae*. *aegypti* in its African centres of origin or diversity. It has been established that the main breeding sites of sylvatic forms of *Ae*. *aegypti* - especially, the predominant sub-species *formosus* - is in forest tree holes, in both West Africa (Sylla et al. 2013) and East Africa (Yalwala et al. 2015). Sampling of these natural water containers for infected eggs and larvae should be the main collecting strategy, although this could be supplemented by the use of artificial containers (Yalwala et al. 2015). Other breeding sites - such as fruit husks - have also been identified (Sylla et al. 2013); offering another sampling niche. Fungal pathogens of the adult mosquitoes are more likely to be found on the understorey vegetation, but this would require an investigative period to determine the most appropriate niches to sample within the forest ecosystem.

It is envisaged that this survey phase would have a short time frame and that the potential of CBC - and thus the longer-term viability of the programme - could be determined within the first year. Once the samples have been processed, identified and catalogued, then the final decision can be made on the feasibility of CBC as a management strategy; obviously, this would be based on the presence or absence of novel, potentially-host specific or coevolved fungal pathogens. If the former prevails, then selected fungi would be assessed for host range, efficacy and pathogenicity, as well as undergoing risk assessments. As detailed previously, the traditional CBC approach is to release hosts infected with the fungus, usually an obligate pathogen, within the target pest populations - whether this be larvae or adults will depend on the spectrum of fungi meeting the selection criteria - and to await natural infection within these populations. Natural dissemination to disease-free populations would follow and thus the establishment of new inoculum sources for future epizootics. There is the distinct possibility that specific, non-obligate (able to grow in vitro) fungal pathogens will be also discovered in Africa. Thus, a two-pronged attack could be considered involving both the classic inoculative strategy as well as a separate inoculative-inundative approach - targeted at the larval stage - whereby a product could be mass-produced and applied as a mycoinsecticide for urban use. This would involve more time - and, potentially, industrial partners - as well as significantly more funding, to develop such a strategy. However, the expectation is that this inundative approach would be relatively short-term and that the fungus would be self-replicating within the urban situation, because of the adaptive traits previously detailed. Development of mass production and delivery techniques will depend firstly on the fungal species selected for use as a mycoinsecticide, but it is expected that the application strategy will be the same as that for conventional insecticides to access the breeding grounds of *Ae. aegypti* within the complex urban landscape. Certainly, the regulatory issues surrounding the exploration and use of components of the microbial biodiversity of the African countries, under the convention of biological diversity, will need to be addressed at an early stage in such projects and, ideally, this should involve scientists from these countries. Likewise, risk-analysis studies - involving both the safety of the agent to be used to non-target arthropods and to humans, including allergenic evaluations if a product were to be developed - will need to be dealt with in order for a formal permit of release to be issued by the relevant authorities in the importing country. The protocol involved is well established and has been tried and tested for exotic fungal pathogens against invasive alien weeds (Evans 2013a), and to a lesser extent against exotic arthropods (Hajek et al. 2007, Hajek & Delalibera 2010).

*In conclusion* - We believe that classical biological control of *Ae aegypti* and, understanding the controlling factors and the natural balance of mosquito populations in Africa that comes with this approach - is just such an innovative vector control strategy that is currently being sought for the management of Zika virus, and related flaviviruses, in the Americas. Moreover, as the resources being allocated to control the pandemic are being assessed, according to their cost and the potential health benefits (Alfaro-Murillo et al. 2016), the cost effectiveness of CBC would far outweigh any of the other, more conventional management strategies presently on offer to decision-makers. In addition to being economically attractive, CBC should also be viewed as a long-term, proactive response rather than an essentially short-term reaction to the crisis, because - as demonstrated by the earlier eradication campaign (Hotez 2016) - the mosquito, as with any other invasive alien species, has the ability to re-invade or bounce back due to its increased fitness in the absence of coevolved natural-enemy pressure. Sustainable or on-going control of the primary vector *Ae*. *aegypti* must be the goal for the current pandemics in the Americas and, potentially, for those in the future when minor or as yet unknown viruses emerge from their forest homes in Africa and spread globally.

Based on the evidence presented, we posit that surveys in sylvatic habitats in both West and East Africa - concentrating on larval breeding sites of *Ae*. *aegypti* in epiphytes and tree crevices, as well as on foliage, for infected adults - will yield a range of coevolved natural enemies. Specifically, entomopathogenic fungi in the orders *Entomophthorales*, *Hypocreales* and *Pezizales* offer the most potential as CBC agents for the safe and sustainable management of *Ae*. *aegypti* in the Americas because of their demonstrated high host specificity; their proven ability to manipulate host behaviour and their plasticity of spore form and function in order to maximise infection. A multinational, multidisciplinary approach will be needed to test the hypothesis.
